# Soil and Mineral Nutrients in Plant Health: A Prospective Study of Iron and Phosphorus in the Growth and Development of Plants

**DOI:** 10.3390/cimb46060312

**Published:** 2024-05-24

**Authors:** Mujtaba Aamir Bhat, Awdhesh Kumar Mishra, Sheezma Nazir Shah, Mudasir Ahmad Bhat, Saima Jan, Safikur Rahman, Kwang-Hyun Baek, Arif Tasleem Jan

**Affiliations:** 1 School of Biosciences and Biotechnology, Baba Ghulam Shah Badshah University, Rajouri 185234, J&K, India; mujtaba@bgsbu.ac.in (M.A.B.); shahsheezma@gmail.com (S.N.S.); mudasirbiotech@bgsbu.ac.in (M.A.B.); saimajan.scholar@bgsbu.ac.in (S.J.); 2 Department of Biotechnology, Yeungnam University, Gyeongsan 38541, Republic of Korea; awdhesh@ynu.ac.kr; 3 Department of Botany, Munshi Singh College, BR Ambedkar Bihar University, Muzaffarpur 845401, Bihar, India; shafique2@gmail.com

**Keywords:** soil, iron, phosphorus, plant growth, productivity, sustainable agriculture

## Abstract

Plants being sessile are exposed to different environmental challenges and consequent stresses associated with them. With the prerequisite of minerals for growth and development, they coordinate their mobilization from the soil through their roots. Phosphorus (P) and iron (Fe) are macro- and micronutrient; P serves as an important component of biological macromolecules, besides driving major cellular processes, including photosynthesis and respiration, and Fe performs the function as a cofactor for enzymes of vital metabolic pathways. These minerals help in maintaining plant vigor via alterations in the pH, nutrient content, release of exudates at the root surface, changing dynamics of root microbial population, and modulation of the activity of redox enzymes. Despite this, their low solubility and relative immobilization in soil make them inaccessible for utilization by plants. Moreover, plants have evolved distinct mechanisms to cope with these stresses and coregulate the levels of minerals (Fe, P, etc.) toward the maintenance of homeostasis. The present study aims at examining the uptake mechanisms of Fe and P, and their translocation, storage, and role in executing different cellular processes in plants. It also summarizes the toxicological aspects of these minerals in terms of their effects on germination, nutrient uptake, plant–water relationship, and overall yield. Considered as an important and indispensable component of sustainable agriculture, a separate section covers the current knowledge on the cross-talk between Fe and P and integrates complete and balanced information of their effect on plant hormone levels.

## 1. Introduction

Agriculture contributes immensely to the growth, development, and economic prosperity of the nation. Considered as the backbone of a nation’s economy, it plays an active role in the reduction in poverty, rural and social development, and food security [1,2]. As the agriculture sector is challenged by different stresses, like drought, salinity, etc., their influences on plant growth and development escalate the problem of food security and agricultural sustainability [3]. Under such circumstances, plants greatly rely on the acquisition of soil nutrients (both macro- and micronutrients) to increase their survival and their productivity is often supplemented by the use of agrochemicals in agricultural practices. Iron (Fe; a micronutrient) and phosphorus (P; a macronutrient) are part of the essential mineral nutrients for the survival of plants [4]. Owing to their relative immobilization and low solubility in soils, their inaccessibility to plants makes them deficient in these essential mineral nutrients with a serious impact on the metabolic machinery of the plant cell. Given the United Nations’ sustainability goal of achieving zero hunger, deficiencies of both Fe and P create serious complications within the plant system that severely impact the yield and quality of the plants, and as such raise concerns at a global scale regarding the availability of food to feed the population. To overcome this problem, the global population has widely become dependent on the use of fertilizers to meet soil requirements in order to achieve agricultural sustainability through increases in crop production and quality. With serious ecological and economic impacts, the use of chemical fertilizers that adversely affect plant health put forth the need to have crops with improved Fe and P requirements toward reducing the use of fertilizers in agricultural practices. Additionally, the growth of plants and their fitness for survival under mineral-deficiency conditions stress the need to have genotypes with efficient mineral nutrient usage to fit the low input of these nutrients in agricultural soils. Keeping this in view, there is a need to have an understanding of the interaction of these mineral elements among themselves, their relation to other mineral nutrients, and, more importantly, the requirement of information that pertains to different signaling pathways operating in response to nutrients in plants. In such a context, the present study aims at summarizing the existing knowledge about these mineral nutrients, the importance of nutrient transporters, the role of siderophores and phosphate-solubilizing bacteria in mineral uptake, cross-talk between them, if any, and information on the signaling pathways that regulate levels of the mineral nutrients, preferably Fe and P, in plants.

## 2. Iron in Plant Health

Iron (Fe), a key micronutrient essential for cellular processes (respiration, photosynthesis, etc.), also functions as a cofactor for the enzymes of vital metabolic pathways, like the ETC (electron transport chain) (oxidoreductases and oxygenases), in the respiration process of plants [5,6,7,8,9]. Fe occupies a key position related to its role: (1) in the synthesis of chlorophyll during photosynthesis [9,10]; (2) as a cofactor of iron–sulfur and heme proteins for the synthesis of phenylpropanoids, oxylipins, and other primary and secondary metabolites, besides being associated with the synthesis of hormones such as gibberellins and brassinosteroids [11,12,13]; and (3) as part of enzymes in nutrient assimilation, such as nitrite and nitrate reductase [14,15]. Plants generally rely on soil Fe content to fulfill their needs as an important component of proteins in chlorophyll synthesis and other metabolic activities. However, only a small portion of it is bioavailable (ferrous form, Fe^2+^) and the rest is present as insoluble matter (ferric form, Fe^3+^), which plants are unable to utilize for various biochemical activities, and as such face Fe deficiency [16,17]. Plants suffer from both deficient (growth on neutral to basic soils), observed mostly in upland regions, and toxic levels of Fe, in soil from lowland agricultural practices where a submerged condition favors the reduction in Fe^3+^-Fe^2+^ (increased solubility) that increases its uptake by plants [18,19]. Under fluctuating environmental conditions, a precise homeostatic mechanism operates in plants that balances nutrient uptake by avoiding surplus storage catalyzing the generation of reactive oxygen species (ROS; preferably hydroxyl radical via the Fenton reaction) [20,21]. As such, optimized Fe uptake is regulated via a transcriptional switch that induces its uptake in response to low internal levels or represses it on account of sufficient Fe stores in plants.

### 2.1. Iron Uptake, Translocation, and Storage in Plants

**Figure 1:**
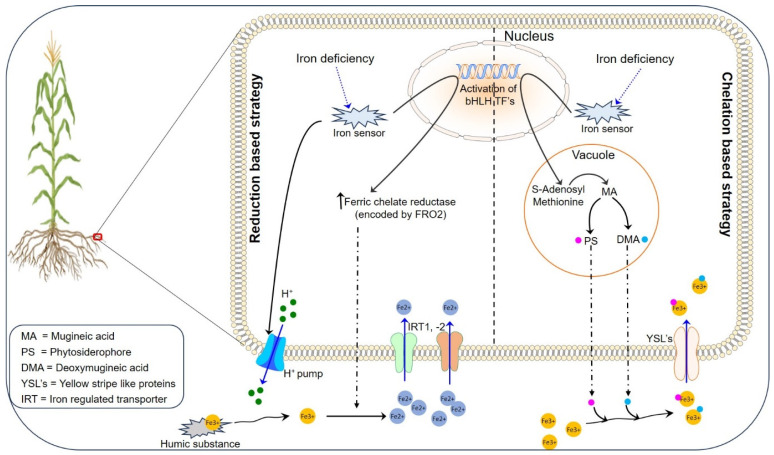
Strategies employed for the uptake of iron at the root surface.

Though abundant in soils, plants have evolved different strategies for maintaining a strict balance of iron by controlling its absorption at the root surface, translocation to aerial parts, compartmentalization for storage, and limiting their remobilization to cope with its deficiency (Figure 1). Plants, being sessile, employ two common strategies: reduction-based (strategy I: non-graminaceous plants, e.g., *Arabidopsis thaliana*) and chelation-based (strategy II: graminaceous plants, e.g., rice) that fine-tunes the mobilization of iron for efficient acquisition at root surfaces [22,23]. The basic components and regulations for the two strategies are discussed below.

#### 2.1.1. Reduction-Based Strategy for Iron Uptake

Strategy I is predominantly utilized by non-graminaceous monocots and dicots, and is based on iron reduction to enhance its uptake under limited conditions. The approach involves the concerted action of different types of plasma membrane proteins of the root epidermis. These proteins are expressed in response to low Fe levels to cope with poorly available soil iron, Fe^3+^ (exhibiting limited solubility and being more prone to precipitation), in the surrounding environment [24]. For this strategy, plants undergo acidification by pumping protons (H^+^) via ATPase AHA2 at the rhizosphere causing the release of Fe^3+^ bound to humic substances and a subsequent reduction to Fe^2+^ by enzyme ferric chelate reductase [10] encoded by FRO2 for its uptake by the IRT (iron-regulated transporter), two genes, IRT1 and IRT2, in *Arabidopsis thaliana* [25,26].

Initially, P-type ATPases play a pivotal role by releasing protons into the rhizosphere, leading to a decrease in soil pH. This acidification enhances the solubility of Fe, with additional contributions from the secretion of solubilizing agents like carboxylates and phenolates. Subsequently, FERRIC-REDUCTION OXIDASES, activated under acidic conditions [27], facilitate the conversion of Fe^3+^ to Fe^2+^. Finally, ZINC-REGULATED TRANSPORTER/IRON-REGULATED TRANSPORTER-LIKE PROTEIN (ZIP) transporters facilitate the uptake of Fe^2+^ into plant roots. In Arabidopsis, H^+^-ATPase 2 (AHA2), FERRIC-REDUCTION OXIDASES 2 (FRO2), and IRON-REGULATED TRANSPORTER 1 (IRT1) encode these three proteins, respectively [24]. IRT1 functions as a high-affinity transporter of Fe, playing a crucial role in both its uptake and distribution from roots to shoots [26,28]. Additionally, NATURAL-RESISTANCE-ASSOCIATED MACROPHAGE PROTEIN 1 (NRAMP1) contributes to the uptake of Fe as a low-affinity Fe^2+^ transporter [29,30]. Recently, a study has unveiled that a complex is formed by FRO2, IRT1, and AHA2 to create a microenvironment with an optimal pH and Fe^2+^ concentration in the rhizosphere. This complex formation helps optimize Fe uptake by preventing the reoxidation of Fe^2+^ into Fe^3+^, a process that may occur due to the presence of oxygen in most soils [31].

The reduction-based strategy was believed to be exclusive to non-graminaceous monocots and dicots. However, subsequent research has demonstrated that grasses also employ this strategy for iron (Fe) uptake. Notably, *Oryza sativa* (rice) utilizes the reduction-based approach by absorbing Fe^2+^ through the activity of OsIRT1, OsNRAMP1, OsIRT2, and OsNRAMP5 [8,32]. In response to the deficiency of Fe, the expressions of OsNRAMP1, OsIRT1, and OsIRT2 are induced, indicating their role in plant Fe nutrition under the conditions of low Fe availability [8]. In contrast, OsNRAMP5, which maintains constitutive Fe^2+^ uptake, does not influence Fe deficiency [32].

Dicotyledonous plants, such as tomatoes, cucumbers, and soybeans, predominantly employ strategy I in response to Fe deficiency, though their specific reactions vary. In the case of cucumber and tomato, Fe-deficient conditions lead to an enlargement of root tips and coarseness near the tips, accompanied by increased root hairs, features characteristic of metastatic cells [33]. These cells actively release a substantial H^+^ concentration, thereby significantly enhancing the root’s Fe^3+^ reduction capability, ultimately improving Fe availability in the rhizosphere. Conversely, soybeans primarily address insoluble Fe^3+^ through the thickening and swelling of root tips, coupled with the accumulation of numerous phenolic substances in the cortex and epidermis [34]. Consequently, certain plants exhibit diverse mechanisms when responding to Fe-deficiency stress. Furthermore, Fe deficiency induces the biosynthesis and signal transduction of ethylene, with ethylene, in turn, playing a role in enhancing Fe transport and distribution in rice [35].

#### 2.1.2. Chelation-Based Strategy for Iron Uptake

Strategy II, prevalent among graminaceous plants, involves Fe^3+^ that undergoes a complex formation with mugineic acid (MA) synthesized from L-methionine with the involvement of nicotinamide synthase (NAS) (catalyzing production of non-proteinaceous amino acid nicotianamine, (NA)), nicotianamine aminotransferase (NAAT), deoxymugineic acid synthase (DMAS) that catalyzes the production of a phytosiderophore (PS), and deoxymugineic acid (DMA), which undergoes hydroxylation to release MA at the root surface [36]. The ferric chelate complex (Fe^3+^-PS) formed in the exterior milieu is transported inside the roots by specific transporters of the yellow stripe-like (YSL) protein family, with its first report in maize, i.e., YS1 [37,38]. To date, 9 variants of MA and 18 YSLs have been reported [8,10]. Though the synthesis of NA is reported as a metal chelator from several plants, only grasses were found undergoing its conversion to PS [22]. As per the reports, OsYSL15 is the major transporter of Fe^3+^-PS in rice, while all other members of the YSL protein family in both graminaceous and non-graminaceous plants are involved in the transportation of metal-Na complexes [39,40]. The efflux of PS DMA into the exterior milieu was recently found to occur through a major facilitator superfamily (MFS) transporter, TOM1 [41]. Additionally, two other MFS member transporters, ENA1 and ENA2, were found to be associated with the transport of NA in plants [41]. AtZIF1 (an analog of ENA1) in *Arabidopsis thaliana* with a zinc-sensitive phenotype and localized on the vacuolar membrane initially thought to be a Zn transporter was recently found to be associated with the accumulation of NA in vacuoles [42]. It is now believed that ENA1 plays a role in metal detoxification via the transportation of NA to the vacuole.

An analogy for rice plants is the occurrence of the Fe^2+^ transporter OsIRT1 that attributes it with the property to take up Fe^2+^ similar to strategy I [43]. However, unlike strategy I, the induction of H^+^-ATPase and ferric chelate reductase was not observed under iron-deficiency conditions. This simply reflects an adaptation to flooded rice where a surplus of Fe^2+^ is present due to reduced oxygen levels [44]. The depleted oxygen levels in soil cause microorganisms to use Fe^3+^ as an alternate electron acceptor, leading to the conversion of Fe^3+^ to Fe^2+^, which is transported into plant roots through the Fe^2+^ transporter, OsIRT1 [45]. The genetic components involved in iron metabolism in rice, particularly *OsIRT1* and *OsIRT2* genes, share similarities with the well-documented strategy I plants. It has been previously reported that ferric chelate reductase activity is induced in iron-deficient conditions, facilitating the reductions in Fe(III) to Fe(II) in growth medium [43]. Connolly et al. [46] observed a decline in the expression of IRT1 and FRO2 mRNA levels as iron concentrations increased from deficient to sufficient levels. Pereira et al. [47] demonstrated the upregulation of the functional gene *OsFRO2* under stringent iron conditions. *OsFRDL1* represents another set of genes accountable for iron efflux transport in rice. Yokosho et al. [48] findings revealed that *OsFRDL1* plays a vital role in iron distribution from the nodes–panicles. Knocking out this gene results in the accumulation of iron in the nodal region deprived of a proper distribution to other plant parts.

#### 2.1.3. Iron Translocation and Storage

During absorption at the root’s surface, Fe undergoes a translocation from the roots to aerial parts having a high iron demand. The transport of Fe inside the shoots takes place through the xylem in a passive mode, until it is unloaded by a specific transporter, and through the phloem in an active mode via the involvement of a specific transport system [49,50]. Inside the plants, the immobilization of Fe takes place as part of its binding to different substrates, such as citric acid, owing to the low solubility in the Fe^3+^ state and being harmful in the Fe^2+^ state [51,52]. Several studies have reported the involvement of different citrate transporters, such as OsFRDL1 (rice) and FRD3 (Arabidopsis), restricted to the xylem-encircling plasma membrane of the root pericycle, for their role of transporting citrate to the xylem [53,54]. Similar to citrate, NA and 2′-deoxymugenic acid are not only involved in the transport of iron through the xylem, but have been reported to be involved in the transport of Fe through the phloem [55,56]. Additionally, YS1 transporters often localized to plasma membranes have been reported to facilitate Fe uptake at the rhizospheric plane and in translocating Fe in the plant body [37,57]. OsYSL15 is regarded as an Fe^3+^-DMA transporter, playing a role in the uptake of iron at the rhizosphere, besides being involved in the translocation of iron following their expression in the shoots and developing seeds [40]. Similarly, OsYSL16 and OsYSL18 are regarded as Fe^3+^-DMA transporters that are involved in transporting Fe to the reproductive organs and allocating it via vascular bundles [58]. In Arabidopsis, vacuole iron transporter-1 (VIT-1) transports Fe into the vacuole [59]. OsVIT-1 and OsVIT-2 (orthologs of VIT-1 in rice) were involved in the translocation of Fe along with zinc (Zn) from leaves to seeds [60]. Natural-resistance-associated microphage proteins (NRAMPs) are recognized as being involved in immobilizing Fe from the vacuoles to cytoplasm for utilization in different plant processes [61]. NRAMP3 and -4 (in Arabidopsis) and OsNRAMP1, -2, and -3 (in rice) are involved in immobilizing iron from the vacuole for its utilization by plants [62,63].

## 3. Phosphorus in Plant Health

Phosphorus (P) (macronutrient) serves as an important structural and functional component of biological molecules like nucleic acids, proteins, ATP, NADPH, vitamins, etc., besides playing an important role in the regulation of signaling cascades occurring in the plant system [64]. It serves as an important component for regulating plant growth, utilized in cellular process, including respiration and photosynthesis, and plays a critical role in the metabolism of carbohydrates and nitrogen compounds [65,66]. Additionally, phosphorus has a significant influence on stress tolerance and a plant’s dependence on mycorrhizal fungi for phosphorus absorption, which is necessary for optimum crop development, yield, and quality [67]. Phosphorus deficiency is among the main factors limiting crop development in tropical and subtropical areas. This deficiency of phosphorus nutrients is caused by extreme temperatures and prolonged rainfall, along with the fixation of phosphorus by aluminum and iron oxides in soil [68]. In the world’s arable land, about 30–40% is affected by phosphorus deficiency [69]. The phosphorus requirement of crops is fulfilled through the use of fertilizers in different agricultural practices. However, ~80% of phosphorus applied as a fertilizer is inaccessible for the majority of crops as inorganic phosphorus rapidly undergoes a complex formation with Ca^2+^ in alkaline soil and with aluminum and iron oxides and hydroxides in acidic soil [70]. As the majority of phosphorus in soil remains inaccessible to plants, it worsens the condition by influencing nutrient availability for crop development. Plants have adapted to changes (biochemical, metabolic, and morphological) in phosphorus availability by increasing the root/shoot ratio, lateral root development, and producing longer and denser root hairs for a larger root surface area, thereby improving its acquisition in response to P limitation [68].

### Phosphorus Uptake, Translocation, and Storage in Plants

**Figure 2:**
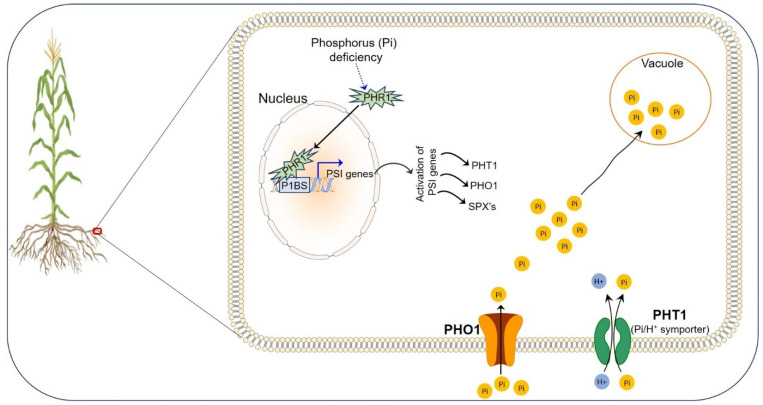
Phosphorus acquisition at the root surface of plants.

Inorganic phosphate (P) is a major source of phosphorus for plants. The concentration of free P ions available for utilization by plants is relatively low (0.01 mmol/L or even less) as the majority of it is present in a complex with metal ions [70]. Though abundant in soil, it is often challenging for plants to absorb it; thus plants have evolved a complex signaling network against fluctuating external phosphate levels and have come up with a selective process of a distinct uptake (transporter) system that improves its availability and internalization for use by the plants [71]. The majority of transporters operating for the uptake of P from soil into the roots includes proton-coupled phosphate transporters (PHTs), SPX domain containing proteins, and sulphate transporter (SULTR)-like phosphorus distributor transporters (SPDTs) (Figure 2).

PHTs, based on subcellular localization, are classified into PHT1 (preferable plasma membrane), PHT2 (localized to chloroplasts), PHT3 (localized to mitochondria), PHT4 (chloroplast, Golgi, and non-green plastids), and PHT5 (localized to vacuoles) [72,73,74]. PHT1 transporters employ the P/H^+^ symporter for transporting P from the soil into the roots and its translocation between cells and tissues. Their number varies across different plant species: Arabidopsis has 9 [75,76], 15 in soybean [77,78], 13 each in rice and maize [72,73,79,80], and tomato having 8 [81]. P uptake via PHT1 relies on the co-transport of 2-4 protons per P [82,83]. PHT2/3/4 facilitates the intracellular transport of P into and out of different sub-cellular organs playing a role in stress response and energy metabolism [74]. These transporters undergo complex formations with each other and, with PHT1 (homomeric as well as heteromeric), play a role in maintaining the homeostasis of phosphate [84]. The vacuole, acting as a depository for the storage of surplus phosphate (~90%), facilitates its mobilization as per the need via PHT5 [73,85]. SPX-domain containing proteins are sub-divided into SPX-major facility superfamily (SPX-MFS), SPX-really interesting new gene (RING), and SPX-EXS (ERD1/XPR1/SYG1) [86], with only SPX-MFS and SPX-EXS reported for their involvement in the transport of phosphate [87].

A potential phosphate transporter, PHOSPHATE 1 (PHO1, a member of SPX-EXS) localized to Golgi is involved in the efflux of cellular P for its loading into the xylem [88]. The Arabidopsis genome contains 10 PHO1 homologs, while in rice, only three have been reported [89,90]. Other members of the SULTR family (SULTR3; 3 and -3;4) are present in rice grains for the accumulation of phosphate [91,92]. Root cells absorb P, loaded into the xylem by the symplastic or apoplastic pathway, transported to the shoot by xylem flow, and dispersed by the phloem across various shoot tissues [64]. Subcellular localization among different organs following transport (root-to-shoot translocation) through the xylem is believed to be mediated by PHO1 [93]. The majority of P entering the roots is absorbed by the symplasm in the outermost cell layers or the root hairs (RHs). In addition to direct absorption using different transporters, an indirect mode facilitated by fungi also contributes to the uptake of phosphate in plants [94,95]. P is released into plant cells by the AMF from pools of the P, which are inaccessible to plant roots [96]. P absorption, translocation, and remobilization are all carried out by P transporters. Current investigations show that their activity and abundance are highly controlled [97,98].

## 4. Roles of Iron and Phosphorus in Plants

In the production of nucleic acids and membranes, phosphorus and iron play a significant role. Fe is therefore necessary for a variety of biological processes, since it is an important component of several essential enzymes, including cytochromes of the ETC (electron transport chain) [99]. In macromolecules, like RNA and DNA, phosphorus connects ribonucleosides. Inorganic phosphate (P) or its organic derivates provide energy to several fundamental biological activities, including photosynthesis and respiration. P plays a critical function in energy transfer processes as a component of nucleotides, such as ATP [99]. Phosphate esters are also used in many different metabolic processes as energy carriers. A vital feature of cellular membranes, phospholipids include the element phosphorus. The transfer and removal of phosphate by enzymes (phosphorylation and dephosphorylation) has a significant impact on how proteins function. Proteins can signal one another through phosphorylation, including a protein’s interactions [99].

In many agricultural plants, iron deficiency is a frequent nutritional disease that leads to low yields and worse nutritional quality. Iron is typically present in aerobic soils in the Fe^3+^ state, mostly as a component of oxyhydroxide polymers with very little solubility. This type frequently falls short of a plant’s requirements [7]. Young leaves that have interveinal chlorosis and stunted root development display visible signs of low iron feeding in higher plants. Iron is necessary to preserve the structure and functionality of chloroplasts and is involved in the production of chlorophyll. In certain conditions, like when the soil is too alkaline (has a high pH), plants face a challenge of absorbing iron because it is mainly in the form of Fe^3+^ chelates in the soil. This difficulty in obtaining enough iron makes it hard for plants in high-pH soils to make and keep chlorophyll, which causes the leaves to become yellow, poor growth, and decreased production [7].

### 4.1. Photosynthesis

Photosynthesis takes place in the chloroplasts, which need a variety of proteins that are encoded in the nuclear genome [100]. While chloroplast to nucleus communication (retrograde regulation) is carefully orchestrated in plants, its molecular mechanisms are little known. Moreover, for chloroplasts to function at their best level, appropriate nutrient accumulation, like iron (Fe), is necessary [101,102]. Fe is present in all electron transfer complexes, including PSI, PSII, ferredoxins, and the cytochrome b6f complex, and is necessary for the synthesis of cofactors such as heme and Fe-S clusters [6,103]. Fe-deficient conditions cause plants to exhibit chlorotic symptoms [104] and have impaired photosynthesis [101,102]. Despite abundant Fe levels, chlorotic leaves may also form in high-phosphorus (P) environments [105], casting doubt on the relationship between Fe concentration and chlorophyll synthesis. According to a study, rice plants growing in environments with a combined Fe and P shortage (FeP) do not display a chlorotic phenotype [106]. The impact of an iron deficit on chlorophyll synthesis and photosystem activity is influenced by the availability of phosphorus [107].

According to Monteiro and Winterbourn, excessive levels of iron stimulated the production of active free ROS (reactive oxygen species), which may have ultimately oxidized the chlorophyll and thus resulted in a drop in chlorophyll concentration [108]. Chlorophyll and carotenoid contents were shown to be lower with higher iron concentrations, according to Arunachala et al. [109]. The regularity of pigment production is the cause of this behavior, and carotenoids are essential metabolites that quench singlet oxygen to protect cellular and subcellular systems from the harmful effects of ROS [110].

### 4.2. Enzyme Activity

According to Asad and Rafique, iron is crucial for plants since it is needed for biological systems and because it is a crucial part of several enzymes, which are important for the biochemical and physiological functions of plants [111]. It takes part in a variety of electron transfer activities and functions as a cofactor of important enzymes of plant hormones [112]. In the metabolism of carbohydrates, the enzyme amylase is involved. According to Hofner, the fact that iron forms complexes with carbohydrates like maltose in plants is the reason why amylase activity is reduced as iron concentrations rise [113]. Together, glutamine synthetase and glutamate synthetase work to convert NH_4_^+^ into amino acids. α-ketoglutarate and glutamine are converted into two molecules of glutamate by the action of glutamate synthase. It is generally accepted that this process is how plants primarily assimilate nitrogen [114]. Because there was less activity, there was also reduced amino acid synthesis, which prevented the plants from assimilating nitrogen. According to Hemalatha and Venkatesan, aspartate amino transferase, amylase, invertase, and glutamate synthase in tea plants were all adversely impacted by excessive iron concentrations [115]. Also, they noted that pigment amounts, like chlorophyll and carotenes, decreased when the iron content dropped. The localized creation of an Fe-polyphenol complex has an impact on polyphenol concentration, which is crucial to tea quality. In graminaceous plants, Bashir et al. investigated the enzyme activity and expression patterns of GR (glutathione reductase) in both iron conditions (iron-deficient and -sufficient) by using two clones of cDNA for cytosolic GR (HvGR2) and chloroplastic GR (HvGR1) [116]. The projected polypeptide lengths for HvGR1 and HvGR2 were 550 and 497 amino acids, respectively, based on their nucleotide sequences. In graminaceous plants, they discovered that GR1 and GR2 genomes were significantly preserved. Both proteins had in vitro GR activity, however HvGR1 had a threefold-higher specific activity than HvGR2. During iron deprivation, HvGR1, HvGR2, and TaGR2 were increased [117]. Furthermore, TaGR1 and HvGR1 were mostly expressed in the shoots, whereas TaGR2 and HvGR2 were mainly seen in the roots. In response to iron deprivation, the activity of GR increased in the shoot tissues of barley, maize, and rice, as well as in the roots of wheat, maize, and barley. The researchers also discovered that rice, wheat, and barley did not present post-transcriptional regulations of GR. The expression of OsGR1 was mostly seen in the roots and calli, rather than the leaves [118]. In graminaceous plants, iron shortage as well as internal iron homeostasis may be influenced by GR.

### 4.3. Metabolism

Iron is a vital micronutrient for nearly all living organisms, owing to its indispensable involvement in fundamental metabolic processes, including DNA synthesis, respiration, and photosynthesis. Additionally, it serves as a cofactor for numerous enzymes, thus activating various metabolic pathways essential for cellular function [7]. Vigani et al. documented that mitochondria serve as the primary sites of energy production in root cells and are notably impacted by deficiencies in iron. Their research highlighted the remarkable functional adaptability exhibited by mitochondria, facilitating the preservation of cellular viability under such conditions [119]. Within mitochondria, a substantial proportion of proteins are metalloproteins, necessitating iron for their proper enzymatic function [120]. Phosphorus (P) plays a pivotal role in plant metabolism by facilitating the synthesis of adenosine triphosphate (ATP), nicotinamide adenine dinucleotide phosphate hydrogen (NADPH), nucleic acids, and phospholipids. These compounds are essential for various aspects of plant development, productivity, signal transduction, and photosynthesis [121,122]. Phosphorus has five key functions in the metabolism of green plants, according to several biochemical investigations on plants conducted over the past fifteen years. 1. Phosphorus may have a role in nitrate reduction in the roots, according to Eckerson [123]. 2. Phosphorus plays a role in the production of starch in the leaves and other green portions of plants. 3. Respiratory functions and phosphorus are somewhat related. 4. Nuclear division is aided by phosphorus. 5. Other researchers believe that phosphorus, which is a component of lipoids and is found in the cytoplasm, helps to facilitate selective absorption by altering the solubility of cytoplasmic membranes or by controlling surface charges [124]. Reduced phosphorus (P) availability has been observed to negatively impact gas-exchange parameters in plants, including the net photosynthetic rate, transpiration rate, and stomatal conductance. Additionally, it has been noted to elevate the intercellular concentration of CO_2_ [125].

## 5. Deficiencies of Iron and Phosphorus in Plants

Phosphorus has gained widely accepted status as part of membrane components, lipids, and in the structural organization of nucleic acids. Playing a vital role in maintaining the structural integrity of macromolecules, its role has also been observed in the processes of respiration and photosynthesis. As per the existing literature, phosphorus (P) deficiency has been demonstrated to exert a significant impact on plants’ physiological parameters. Specifically, it leads to a substantial reduction in the dry weight (DW) of both shoot and root tissues in lettuce and tomato, along with a decrease in leaf numbers observed in lettuce, Chinese milk vetch, alfalfa, tomato, and marigold [126]. Across various plants, such as sunflower, rice, and maize, P deficiency induces a noteworthy decline in both the net photosynthesis rate and PSII reaction center’s efficiency for energy capture [127]. This deleterious effect on the photosynthesis rate due to P deficiency has also been documented in diverse crops, including sugar beet [128], soybean, tobacco, *Zizania latifolia*, oat, sheep grass, barley, and tea [129,130,131]. Consequently, the insufficiency of phosphorus compromises the grain yield or overall crop production [125,127]. Moreover, short-term phosphorus deprivation not only leads to a reduction in phosphorus concentration, but also results in the diminished content of total chlorophyll and carotenoids in tomato seedlings [132].

Phosphorus (P) deficiency is known to perturb metabolic processes and carbohydrate translocation, including organic acids and soluble sugars [133,134]. The response to P deficiency often involves an augmented accumulation of carbohydrates, particularly sucrose, in the leaves of various species of plants [135,136]. This accumulation is attributed to reduced sink demand and constrained leaf expansion under conditions of P starvation [137]. Notably, the secretion of organic acids represents a pivotal low-P response in plants, facilitating the dissolution of soil P through acidification and complexation. This mechanism contributes to diverse levels of low-P tolerance in crops [138,139]. Some species, such as tea [140], white lupin [141], barley, soybean [138], and Chinese fir [139] have been observed to exhibit an induced exudation of organic acids under low-P conditions. Moreover, investigations suggest that the internal metabolism of organic acids may also play a role in low-P responses, exemplified by significantly elevated concentrations of citric, malic, and succinic acids in the roots of low-P alfalfa [142]. The deficiency of P as compared to other abiotic stresses inevitably triggers an escalation in the generation of ROS as byproducts of photosynthesis. A feedback inhibition mechanism, orchestrated by the accumulation of sugars, results in the diminished utilization of the electron transport chain [143].

The deficiency of phosphorus in plants proceeds with a change in the pigmentation (dark-green coloration) of the leaves and stunted plant growth. Phosphorus deficiency leads to the excessive production of anthocyanins that change the coloration of leaves to slightly purple (not correlated with leaf chlorosis). Its deficiency causes a delay in the maturation process of plants and in inducing symptoms of slender-like stem, as observed for the deficiency of nitrogen and the development of small spots of dead tissue (often referred to as necrotic spots). Considering its essentiality for plant growth and development, its requirement is directly correlated with the enhancement in the yield of different crops [144,145]. Its deficiency is generally addressed through the application of phosphate-based fertilizers that complement soil nutrients and make it available for use by plants [146].

Approximately one-third of globally cultivated land consists of calcareous soils, and these soils are a major contributor to iron (Fe) deficiency, which is prevalent in about 40% of all soil types [147]. The manifestation of Fe deficiency gives rise to diverse metabolic irregularities, leading to anomalies in chloroplast structure and morphology, diminished chlorophyll levels, reduced photosynthetic rates, and compromised respiratory functions in plants. These adverse effects collectively result in a substantial reduction in both the yield and quality of crops [148,149]. Consequently, iron deficiency poses a widespread challenge impacting agricultural productivity. In plants, iron is required for the assembly and maintenance of chlorophyl structure. Having such important functions, its deficiency often causes the chlorosis of the leaves. The symptoms of iron deficiency progress from intravenous chlorosis to the chlorosis of whole leaves (for prolonged deficiency) that turns the color of the leaves to white. The formation of insoluble iron decreases its mobility that increases its precipitation in older leaves. Iron undergoes a complex formation with the iron-binding protein, phytoferritin, as observed in the leaves and other plant parts [150].

## 6. Bacteria-Mediated Uptake of Fe and P

Bacteria mediates the uptake of mineral nutrients, especially Fe and P, through the production of siderophore (for Fe) and solubilization of P reserves for uptake by the plant system.

### 6.1. Siderophores in Fe Uptake

Several studies have demonstrated the efficacy of siderophore-producing rhizobacteria in promoting plant growth. Examples of such bacteria include *Bacillus licheniformis*, *B. subtilis*, *B. coagulans*, *B. circulans*, *Pseudomonas fluorescens*, *P. koreensis* [151], *Bacillus cereus*, *Pseudoalteromonas tetraodonis*, *Micrococcus aloeverae*, *Psychrobacter pocilloporae*, *P. weihenstephanensis* [152], *P. aeruginosa* [153,154], *Bacillus*, and *Rhodococcus*, and Enterobacter genera [155] and Pseudomonas sp [156]. Additionally, *Pantoae cypripedii* [157], *Pantoae dispersa* [158], *Bacillus megaterium* [159], and *Kosakonia radicincitans* [160] have also been identified as contributors to plant growth promotion through siderophore production.

Siderophores play a vital role in numerous physiological processes in plants, including respiration, bioremediation, photosynthesis [161,162], promoting the growth of plants, and aiding in the phytoremediation of heavy metals [151,163]. These compounds, produced through both non-ribosomal peptide bonds and multi-dentate iron-chelating compounds, demonstrate the ability to dissolve and bind various compounds (organic and inorganic) in soil [164,165]. Siderophore-producing bacteria primarily release proteins (like permeases and ATPases), which are iron-binding, facilitating the chelation of Fe^3+^ (ferric iron) and subsequent transport of Fe^3+^ ions in Gram-positive bacteria through the cell membrane [166]. In contrast, the transport of Fe^3+^ (ferric iron) in Gram-negative bacteria employs a sophisticated mechanism involving various enzymes, outer membrane receptors, periplasmic binding proteins, and cytoplasmic membrane proteins for ferric iron (Fe^3+^) transportation, ultimately making Fe^3+^ available to plants [167].

Plant growth stimulation and health protection are two functions of siderophores. Using radio-labeled ferric siderophores as a plant’s only source of iron has shown the advantages of microbial siderophores [156]. Further evidence for the siderophores’ importance in plant nutrition comes from the lack of indications of an iron shortage, such as chlorosis, and the relatively high iron content in the roots of plants grown in non-sterile soils as opposed to plants produced in sterile environments [168]. In plants that were not infected with the siderophore-producing Pseudomonas strain GRP3 and cultivated in environments with a limited iron supply, the injected plants displayed less chlorotic signs and more chlorophyll [169]. When nickel is introduced to *A. thaliana*, the plants were inoculated with two mutants of the *Pseudomonas putida* ARB86 strain—one overproducing siderophores, and the other lacking siderophore synthesis—the symptoms caused by the metal were relieved to the same degree in plants inoculated with the wild type and both mutants, indicating that siderophore independence is involved, in this case, of alleviating Ni toxicity [170]. Similarly, two strains of bacteria that produce siderophores decreased the amount of zinc that *Salix caprea* (willow) absorbed, indicating that siderophores of bacteria may bind to heavy metals in the soil and prevent plants from taking up those metals. However, improvements in zinc and copper absorption in willow infected with a strain of Streptomyces incapable of producing siderophores emphasize the significance of other physiological characteristics for heavy metals [171].

### 6.2. Phosphate Solubilization

Phosphate-solubilizing bacteria (PSB) play a pivotal role in fostering a symbiotic relationship with plants by contributing to the efficient utilization of phosphorus, an essential nutrient for plant growth [172]. Phosphorus is often present in soil in insoluble forms, primarily as phosphate minerals or organic compounds, making it challenging for plants to access [173]. In response to this challenge, PSB has evolved mechanisms to solubilize these otherwise inaccessible phosphates, rendering them in a form that plants can readily absorb (Table 1).

One of the key functions of PSB involves the secretion of enzymes, such as phosphatases, which break down complex organic and inorganic phosphates into simpler, soluble forms [192]. This solubilization process occurs in the rhizosphere, the region surrounding plant roots, where phosphate-solubilizing bacteria are often found [193]. By converting insoluble phosphates into accessible forms, these bacteria aid in the enhancement of phosphorus availability for plants [194]. The intimate association between phosphate-solubilizing bacteria and plant roots is crucial for the success of this relationship [195]. PSB can colonize the rhizosphere and adhere to root surfaces. This colonization facilitates direct interactions between the bacteria and plant roots, enabling a more efficient exchange of nutrients [172]. As a result, plants can absorb solubilized phosphorus more effectively, supporting critical physiological processes, like energy transfer, nucleic acid synthesis, and root development [196]. Furthermore, the impact of phosphate-solubilizing bacteria extends beyond nutrient solubilization. Some strains of these bacteria exhibit plant growth-promoting traits, producing substances like phytohormones and organic acids. In plants, these compounds contribute not only to enhanced nutrient absorption, but also improved stress tolerance [197]. The presence of phosphate-solubilizing bacteria can positively influence the overall health and resilience of plants, particularly under conditions of nutrient stress. In the broader context of soil health, the activities of phosphate-solubilizing bacteria contribute to nutrient cycling. By solubilizing phosphates, these bacteria facilitate the recycling of phosphorus within the soil ecosystem, benefiting a diverse range of plants. This nutrient cycling is fundamental for maintaining soil fertility over the long term [198]. As a practical application of this symbiotic relationship, biofertilizers containing phosphate-solubilizing bacteria have been developed. Farmers can use these biofertilizers to enhance the fertility of soil and promote the growth of plants in an environmentally sustainable manner [199].

## 7. Toxicity Effects of Excess Fe and P on Plants

The inherent redox properties of Fe enable it to serve as both an electron donor and acceptor in cellular environments. In the presence of unsequestered iron within the cell, it can catalyze the transformation of hydrogen peroxide into free radicals through Fenton chemistry. These free radicals, possessing unpaired electrons, exhibit high reactivity and can induce oxidative damage to diverse cellular components. Cumulative oxidative stress inflicted by free radicals may culminate in cellular demise [200]. Enzymes involved in the transport of electrons need iron as a key component in redox reactions (e.g., Fe-S proteins and cytochromes). Moreover, Fe is a component of proteins (non-heme iron) that are essential for respiration, photosynthesis, and N_2_ fixation [201]. One-third of the world’s arable land is affected by iron restriction, which is a significant issue for agriculture. Several photosynthetic components, such as ferredoxin (Fd), the Fe-S protein, involved in crucial oxidoreductive processes of chloroplasts, suffer from iron deficiency, contributing to this loss [202]. During the electron transfer process, it is reversibly oxidized from Fe^2+^ to Fe^3+^ in this function. Interveinal chlorosis, a symptom of iron insufficiency related to Mg deficiency, is one of its defining signs [203]. Iron deficiency typically causes young leaves to exhibit interveinal chlorotic signs as well as poor root development, and when severe, the shortage results in stasis, growth retardation, and mortality [204]. Phosphorus toxicity in plants is a significant concern that arises when there is an excess of phosphorus available in the soil or when plants are subjected to high levels of phosphorus fertilization. This condition can have detrimental effects on plant health and productivity. Phosphorus toxicity often manifests through visible symptoms on plant leaves [205]. These symptoms can include necrotic spots or patches on the leaves, particularly along the leaf margins. In severe cases, the leaves may become entirely necrotic and die off. Additionally, plants may exhibit reduced growth, including the stunted growth of both the shoots and roots. Different plant species exhibit varying degrees of tolerance to phosphorus toxicity. Some species are highly sensitive and may show severe symptoms, even with relatively low levels of phosphorus, while others are more tolerant and can withstand higher concentrations of phosphorus without significant damage [206].

### 7.1. Germination and Plant Growth

Iron (Fe) toxicity poses a significant threat to seed germination. In *Vigna radiata* (green gram), Verma and Pandey, highlighted that germination peaked at a 100 µM Fe supply, but a decline was observed between 200 and 300 µM Fe applications [207]. Similarly, an increase in Fe toxicity led to a reduction in seed germination in wheat and cowpea [208,209]. Consistent findings were reported by El Rasafi et al., [210] in common bean (*Phaselous vulgaris*) and wheat (*Triticum aestivum*), reinforcing the negative impact of elevated Fe levels on seed germination. Additionally, Nozoe et al. [211] noted an inhibition of the germination of rice weed (*Echinochloa oryzicola* cv. Hoshino yume) and cockspur grass (*Echinochloa crus*-galli) with the application of 100 mg L^−1^ of Fe^2+^. Their examinations of rice and weed tolerance suggested the potential use of Fe for weed control fertilization amendments. Furthermore, Rodrigues Filho et al. found that an increased Fe^2+^ content in the substrate significantly reduced radicle formation [212]. A dose of Fe^2+^ (50 mg/L) resulted in the increased peroxidation of lipid, particularly in the radicle zone, causing growth stunt and decreased dry weight production [213]. Inhibited radical growth has severe consequences for various processes related to development, including the loss of radicle hairs and volume reduction [19]. Phosphorus toxicity proceeds with oxidation damage to the macromolecular structure. Lipid peroxidation, which is the oxidative degradation of lipids, may occur due to the accumulation of reactive oxygen species (ROS), further damaging plant tissues [205]. The mechanisms underlying phosphorus toxicity involve disruptions in various physiological processes in the plant. Moreover, high levels of P can inhibit the activity of certain antioxidant enzymes, such as Cu/Zn-type superoxide dismutase, which are essential for scavenging ROS and protecting plant cells from oxidative damage [205]. One significant consequence of phosphorus excess is the accumulation of phytic acid in plant cells. Phytic acid is a phosphorus-rich compound that forms when plants store excess phosphorus in the form of phytate.

### 7.2. Plant–Water Relation and Yield

The translocation of Fe occurs along the xylem transpiration stream [214]. Hence, closing the stoma might be viewed as an avoidance strategy to reduce its absorption and translocation. Many investigations have found that elevated Fe toxicity results in reduced stomatal conductivity [215,216]. In plants (tolerant and sensitive), this diminishes stomatal conductance as soon as Fe is applied [217]. Yield decrease and the severity of Fe have some relationships. This connection is influenced by both the cropping scheme and the season. Sahrawat (2010) estimates that typical yield losses brought on by Fe toxicity vary from 12% to 35% [218]. Rice yield loss occurs above a critical toxicity of Fe around 500 mg/kg (dry weight of leaves) [219,220]. Rice production loss is closely related to the soil solution Fe^2+^ concentration and cultivar tolerance level [221]. A maximum yield loss was noted at a 500 µM Fe supply [207]. In the condition of Fe toxicity, the formation of both reproductive and vegetative tillers was reduced. Becker and Asch discovered spikelet sterility, maturity, and delayed flowering in the range of 20–25 days; they also saw no blooming of sensitive cultivars [214]. Fe poisoning at early reproductive and late vegetative stages resulted in less panicles per hill [222]. The critical threshold of phosphorus concentration associated with a decline in plant yield varies among species. This threshold is influenced by factors such as the plant’s nutrient requirements, growth habits, and environmental conditions. Reported critical phosphorus concentrations range from 0.26% to 1.00%, depending on the specific plant species and growing conditions [223,224]. Excessive levels of inorganic phosphorus, particularly phosphate ions (Ps), can interfere with photosynthesis. This interference may occur through several pathways, including a reduction in the activation of Rubisco. The accumulation of phytic acid can have several negative effects on plant metabolism. It can interfere with the availability of essential metals within the plant, such as iron and zinc, by forming insoluble complexes with these metals. This, in turn, can impair various metabolic processes and reduce the overall health and productivity of the plant.

### 7.3. Nutrient Uptake

As per various studies, a heightened iron (Fe) concentration in the soil causes in chemical interactions with Fe^2+^, resulting in the diminished uptake of essential nutrients [225]. Researchers have made a distinction between “pseudo-Fe toxicity” and “real Fe toxicity,” the latter being induced by elevated Fe^2+^ levels, impeding the absorption of some other crucial nutrients. The phenomenon is evident in rice plants, where an increased concentration of Fe^2+^ in the soil leads to reductions in calcium (Ca), potassium (K), manganese (Mn), phosphorus (P), and magnesium (Mg) contents in the shoots [226]. This occurs due to the increased content of metal in the root, stem, and lamina. Olaleye and Ogunkunle, argue that Fe poisoning amplifies the nutritional demands for phosphorus (P) and potassium (K) to alleviate stress reactions [227]. Additionally, plants receiving 10 mg L^−1^ of Fe experienced an increase in iron (Fe) content and reduction in potassium (K) content in both the shoots and roots [228]. Soils deficient in potassium (K) often exhibit high or hazardous levels of iron (Fe) [225]. Furthermore, in zinc-deficient soils, an excess of iron (Fe) not only leads to a deficiency in calcium (Ca), magnesium (Mg), phosphorus (P), and potassium (K), but also exacerbates the antagonistic relationship between iron (Fe) and zinc (Zn) [229]. This intricate interplay emphasizes the far-reaching consequences of iron toxicity on nutrient dynamics in the soil–plant system. The effects of phosphorus toxicity can be influenced by the availability of other nutrients in the soil [205]. High levels of nitrogen or potassium, for example, may help alleviate the toxic effects of excess phosphorus to some extent by promoting plant growth and mitigating nutrient imbalances [230]. Compared to Fe, excessive phosphorus can interfere with the uptake of other essential nutrients, such as iron, zinc, and manganese, leading to nutrient deficiencies [4]. High phosphorus levels can inhibit root growth and development, reducing the plant’s ability to absorb water and nutrients from the soil [231]. A high phosphorus concentration can disrupt various metabolic pathways in the plant, affecting processes such as photosynthesis, respiration, and hormone regulation [126].

## 8. Iron–Phosphorus Homeostasis in Plants

It has long been known that plants may interact with macronutrients and micronutrients, and this interaction is somewhat understood. The relationships between Zn, P, and iron (Fe) homeostasis at molecular and physiological levels are therefore highlighted in this article. Crop species have shown evidence of the connection between the homeostasis of two nutrients. In plants, the relationship of P and Fe homeostasis is largely recognized. During Fe-deficit conditions, P acquisition in both the shoots and roots is encouraged, while in contrast, in the plants, P deficiency greatly enhances Fe availability [103,232,233].

Recent plant research has unveiled intricate interactions between macronutrients and micronutrients, particularly highlighting the complex tripartite relationship between Fe and P homeostasis in Arabidopsis [234]. The interplay of P and Fe shortages governs iron transport in rice [106]. The tripartite interaction between Fe and P homeostasis is highlighted in this study as a factor in increased plant survival and fitness.

Molecular and physiological relationships have been established between Fe and P nutrition in well-studied model systems in rice and Arabidopsis [235,236]. However, limited knowledge exists regarding these interactions in mycorrhizal plants. Studies on edible crop species in mycorrhizal plants have indicated a negative association between Fe and P absorption [237,238]. Mycorrhizal plants tend to acquire more Fe under a low-P supply, while host plants acquire less Fe under P circumstances during arbuscular mycorrhizal (AM) symbiosis. Notably, the Fe content in straw significantly increased during AM symbiosis with a low supply of P, indicating that mycorrhized rice reduces the amount of Fe transferred to the shoots at a high P level [238]. Similar detrimental effects on Fe concentration were observed in maize treated with P fertilizers [239]. Consequently, in mycorrhizal plants, the accumulation of Fe is adversely affected by the application of high P to soil [240]. While these studies shed light on the impact of a high level of P on Fe absorption during AM symbiosis and transport in maize and rice, the reciprocal effect on P nutrient uptake by Fe treatments in mycorrhizal plants remains an underexplored area.

### 8.1. Fe-P Cross-Talk

Studies on the nutrient status of plants have revealed the strong physio-biochemical interaction of Fe and P occurring in plants [241]. On the rhizospeheric plane, the formation of the Fe-P complex renders Fe and P unavailable for uptake by the roots for its utilization in different cellular processes in the plant. For plants growing in limited-Fe and surplus-P conditions, a special uptake system for Fe is activated, while, in vice versa conditions, the accumulation of Fe accompanied by aluminum has been reported [103,233,235]. Other mechanisms of plant tolerance to P deficiency include an increase in the solubility of P for uptake at the root surface following the release of organic acids via transporters of citrate and malate [242]. Having a high affinity for Ca^2+^, Al^3+^, and Fe salts, organic acids easily replace P from insoluble precipitate for acquisition by the roots [243]. The interaction of Fe with P progresses with a reduction in its translocation to the aerial parts of the plants [244,245]. A similar pattern has been observed on the leaf surface, where chlorosis mediated by a high level of P occurs, irrespective of the presence of significant amounts of Fe [105]. Inside the seeds, the storage of Fe occurs in the vacuoles following the formation of the inositol hexakisphosphate-Fe complex [246]. This all shows that P acts as an Fe chelator in plants that affects its availability during changes in P homeostasis; therefore, there are hints of similar types of interactions between Fe and P that maintain their optimum levels in the shoots as well as seeds.

In arabidopsis, the availability of Fe plays a vital role in changing root architecture following the induction by a deficiency of P [235]. The reduction in the level of P results in the inhibition of root elongation that is restored under Fe-deficient conditions without any interference in P availability. Contrary to this, an increase in Fe accumulation was reported in the shoots and roots of Arabidopsis following growth under P-starved conditions [235,247]. In plants, a higher Fe concentration observed under P-deficient conditions is attributed to the expression of the Fe-responsive genes [232].

In phosphate-deficient plants, a transcriptomic analysis revealed that the expression of genes associated with the regulation of excess iron is enhanced, while a decrease in gene expression associated with iron deficiency was observed [247,248]. The expression of the gene *AtFER1* (encode ferritin protein) that regulates iron storage was induced in plants with a P deficiency. However, this regulation of the expression of *AtFER1* was found to be independent of the requirement of cis-acting element iron-dependent regulatory sequences (IDRSs) found in the promoter region [249]. Irrespective of iron availability, the expression of the proteins associated with iron storage is mediated by two members of the MYB transcription factor family (PHL1 and PHR1) through binding with the cis-regulatory element, located in the promoter region (P1BS) of the *AtFER1* gene [249]. In response to excessive Fe, the upregulation of the expression of *AtFER1* remains unchanged in single (phr1) as well as in double mutants (phl1 and phr1) [249]. The deficiency of phosphorus (P) was observed by inhibiting the expressions of *FRO3, -6, IRT1, -2*, and *NAS1* genes in wild-type plants. Conversely, in phr1 × phl1 double mutants, these genes were found to be upregulated [103,233,250]. These results underscore the vital role of PHL1 and PHR1 in preserving the homeostatic interaction between phosphorus (P) and iron (Fe) in plant systems.

The core transcription factors PHR1 and PHL1 control P transporters’ expression (PHO1 and PHT1) under P-deficiency conditions via signaling across the PHR1-miR399-PHO2 signaling cascade. P deficiency promotes Fe accumulation at the root surface via modulation signaling across the STOP1 cascade (PDR2-LPR1-STOP1-ALMT1) [251,252]. Transcription factors PHR1 and PHL1also help in maintaining Fe homeostasis via regulating the expression of Fe transporters (NAS3, FER1, and YLS8). Under conditions of Fe sufficiency, IDRS (cis-acting element) plays an important role in maintaining Fe homeostasis via upregulation in the expression of ferritin genes (FER2, FER3, and FER4) with the simultaneous inactivation of FIT to inhibit the expression of genes related to Fe uptake (IRT1 and FRO2) [253]. Following the interaction of bHLH IVc and bHLH121, a deficiency of Fe was found to exert a positive regulatory effect on bHLH Ib and FIT expressions [254]. The interaction of FIT and bHLH Ib in turn induces the expression of genes (IRT1 and FRO2) and P transporter genes, such as PHO1, necessary for P homeostasis [26,253]. Taken together, proteins such as PHL1, PHR1, PHO1; PHO1;1, H1, STOP1, FER1, and MPK6 extend help in integrating the signals for Fe and P as part of their cross-talk in plants.

### 8.2. Hormones concerning Fe and P in Plants

Phytohormones, being small endogenous molecules, play a crucial role in eliciting effective defense responses against stresses (biotic and abiotic) in plants. Beyond their involvement in defense signaling, these phytohormones serve as growth regulators, displaying various physiological and development processes. Phytohormones, including auxins (AUXs), gibberellins (GAs), cytokinins (CKs), ethylene (ET), brassinosteroids (BRs), salicylic acid (SA), abscisic acid (ABA), and jasmonic acid (JA), respond to stress through antagonistic and synergistic actions, commonly referred to as signaling cross-talk. This intricate coordination enables plants to respond adaptively to developmental and environmental cues. While phytohormones are well-established for their roles in both development and defense, specific hormones, like auxins, brassinosteroids, cytokinins (CKs), and gibberellic acid (GA), are particularly associated with the growth and development of plants [255,256,257]. Despite their primary impact on growth, these phytohormones also play a regulatory role in modulating plant defense responses [258,259,260]. In the context of systemic resistance pathways, phytohormones like jasmonic acid (JA), ethylene (ET) [261], salicylic acid (SA) [262], and abscissic acid (ABA) are notably involved [263]. When phosphorus (P) or iron (Fe) are deficient, auxin and ethylene have been linked to the development of roots [264,265,266,267]

In Arabidopsis, an increase in auxin synthesis is induced by a deficiency in iron, leading to elevated expressions of *FIT* and *FRO2* genes. Furthermore, the transcription of these genes is stimulated by the application of exogenous auxins [268]. An increase in ethylene production was observed in cucumber (*Cucumis sativus* L.), peas (*Pisum sativum* L.), and tomato (*Lycopersicon esculentum* Mill.), when there was a deficiency in iron [269]. Treating Arabidopsis and tomato with ethylene precursor ACC (1-aminocyclopropane-1-carboxylate) resulted in the heightened expression of *FRO*, *FIT*, and *IRT* genes. Conversely, in plants grown under Fe-deficient conditions, ethylene inhibitors’ application led to the repression of these gene expressions [270]. Further investigations have shown that iron deficiency triggers an increased expression of genes involved in ethylene signaling (AtCTR1, AtEIN2, AtEIN3, AtETR1, AtEIL1, and AtEIL3) and ethylene synthesis (*AtACS4*, *AtACS6*, *AtACS9*, *AtSAM1*, *AtSAM2*, *AtACO1*, and *AtACO2*) [271]. Jasmonic acid is a negative regulator of iron-deficiency response [272]. Arabidopsis subjected to the conditions of iron-deficiency and methyl jasmonate treatment (100 μM) exhibited a reduced expression of genes (*AtIRT1*, *AtFRO2*, and *AtFIT*). Similarly, when JA (20 μM) was applied to *Medicago truncatula*, the expressions (*MtFIT, MtFRO3*, *MtbHLH38,* and *MtbHLH39*) of iron-deficiency response genes were reduced, which in turn caused a decrease in the chlorophyll content [273]. In contrast, the application of salicylic acid (SA) to Arabidopsis increased the expression of genes (*AtbHLH38* and *AtbHLH39*), pivotal for establishing the response to iron deficiency [274]. Nevertheless, in *M. truncatula* exposed to 100 μM of SA, the gene expression related to iron deficiency decreased [273]. Interestingly, the content of chlorophyll increased by the treatment of peanut plants (*Arachis hypogaea*) with SA under both iron-sufficient and -deficient conditions [275], presenting a contrasting result compared to *M. truncatula* [273].

The expansion of the absorptive surface area in response to P and Fe deficiencies is achieved through the increased length and frequency of root hairs in Arabidopsis. These extra root hairs frequently appear across a tangential wall of underlying cortical cells, which is a location typically inhabited by non-hair cells under normal circumstances. Through phenotypic investigations of hormone-related Arabidopsis mutants and the application of chemicals that interfere with transport, or perception, hormone production, the role of ethylene and auxin in the growth of the root’s epidermal cell under P and Fe deprivation was inferred. The application of the auxin analog (2,4-D) or ethylene precursor (1-aminocyclopropane-1-carboxylic acid) notably increased root hair density, resembling the phenotype of ethylene-overproducing mutants. Further investigations revealed that the application of hormone antagonists and hormone insensitivity inhibited the initiation of extra root hairs induced by the deficiency of Fe. However, these measures did not counteract the formation of additional hairs in response to P deprivation. A model is proposed, outlining potential pathways for alterations in root epidermal cell patterning induced by environmental stress.

Ethylene (ET) and nitric oxide (NO), with other signaling substances, have been implicated in the development of most morphological responses to Fe deficiency. Pioneering studies by Romera’s group delved into the investigation of ET/NO interactions in this regulatory process [269]. ET and NO also contribute by regulating the cell wall composition and the dynamics of endodermal cell suberization [271,276]. Under Fe-deficient conditions, a naturally occurring polyamine (putrescine) is synthesized at an increased rate, which causes an NO burst that serves as a positive regulator for Fe solubilization that is attached to cell walls. Notably, the advantages of Fe utilization for the cell wall of putrescine is compromised in mutants of NO synthesis, including the *noa1* mutant (NO-associated 1) and *nia1nia2* mutants (nitrate reductases 1 and 2) [277]. Furthermore, this positive effect was absent in the Xyloglucan endotransglucosylase/hydrolase 31 mutant (*xth31*), the hemicellulose production mutant [277], and the reduced putrescine (Put) content mutant *adc2* [278]. Lei et al. demonstrated that genes PT1 and PT2-encoding P transporters are expressed less regularly in *etr1* and *ein2-5* (ET-insensitive Arabidopsis mutants), whereas the constitutive ET signaling mutant *hps2* displays enhanced responses to P deficiency [279]. ET has also been implicated in the regulation of *PHT1;5* [280,281]. In plants like *Medicago falcata*, the evidence suggests the involvement of ethylene (ET) in regulating phosphorus (P)-deficiency responses [282]. The expression of P transporter-encoding (*MfPT1* and *MfPT5*) genes as well as the acid phosphatase-encoding (*MfPAP1*) gene was strongly suppressed by ET synthesis inhibitors, including cobalt (Co^2+^) and aminoethoxyvinyl glycine (AVG). On the other hand, applying the ACC to plants increased the expression of these genes, especially in plants that were cultivated in settings where P was adequate [282].

Cytokinins play a crucial role in the regulation of phosphorus (P)- and iron (Fe)-deficiency responses [283]. Their involvement in nutritional signaling responses was initially recognized in the early 1980s when studies demonstrated a decrease in cytokinin levels under conditions of P and nitrogen (N) deficiencies [284]. In the context of P deficiency, the exogenous application of cytokinins has been shown to repress the expression of P-acquisition genes [285]. More recently, a similar repressive effect of cytokinins was observed for the expression of Fe-acquisition genes, including *IRT1*, *FRO2*, and *FIT* [286]. These findings underscore the presence of shared components in the responses to both Fe and P deficiencies, highlighting the significance of cytokinins alongside ethylene in these regulatory processes.

## 9. Conclusions and Future Perspectives

Plants, being sessile, are exposed to variable stresses, including soil nutrient availability, exposure to metals, etc., which exerts significant pressure on the survival and overall development of plants. Influenced by root architecture, plants are easily exposed to nutrient stress, such as the presence of a reduced amount of minerals, such as Fe, P, etc., influencing the growth and development parameters in their life cycle. The imbalance of mineral nutrients is rectified by increasing the use of inorganic and organic fertilizers that amend the availability of nutrients for uptake by plants. The use of fertilizers requires high energy, a large capital base, and a significant amount of effort, and mainly focuses on increasing productivity with little or no concern for damage to the ecosystem. As mineral nutrients play a pivotal role in the nutrition of plants, unraveling the intricacies of the transporters and pathways associated therein would help in understanding the significance of mineral nutrient cross-talk for efficient nutrient management. It highlights the need to unlock the avenues for crop improvement by undertaking integrative studies toward developing stress-resilient crop varieties. Although the imbalance of nutrients, specifically minerals, are adjusted by increasing the biomass of organs involved in acquiring scarce resources, they often regulate these imbalances through the development of effective strategies toward maintaining nutrient homeostasis. To this end, stable strategies and methodological procedures are needed to be undertaken, not only to minimize environmental challenges, but to increase plant productivity and profitability that would boost the agriculture-based economy.

## Figures and Tables

**Table 1:** Phosphate-solubilizing microorganisms, production of organic acids, and their ecological niches.

Phosphate-Solubilizing Microorganisms	Predominantly Produced Acids	Ecological Niche	References
*Escherichia freundii*	Lactic acid	Soil	[174,175]
*Penicillium* sp., *Aspergillus niger*	Citric acid, succinic acid, oxalic acid, glycolic acid, gluconic acid, lactic acid	Soil	[176,177]
*Bacillus subtilus*, *Bacillus megaterium*, *Pseudomonas* sp.	Lactic acid, malic acid	Soil rizoshpere	[177]
*Arthrobacter* sp., *Bacillus* sp. *Bacillus firmus* B-7650	Citric acid, lactic acid	Cowpea and wheat rhizospheres	[178]
*Chaetomium nigricolor*, *Penicillium* sp., *Aspergillus* sp.	Oxalic acid, succinic acid, citric acid, 2-ketogluconic acid	Lateritic soil	[179,180]
*A. foetidus*, *A. japonicus*	Citric acid, tartaric acid, succinic acid, oxalic acid, gluconic acid	Indian rock phosphate	[175]
*P. radicum*	Gluconic acid	Wheat rhizosphere	[181]
*Enterobacter agglomerans*	Citric acid, oxalic acid	Wheat rhizosphere	[182]
*Enterobacter aerogenes*, *E. asburiae*, *E. taylorae*, *Penibacillus macerans*, *Vibrio proteolyticus*, *Kluyvera cryocrescens*, *Xanthobacter agilis*, *Pseudomonas aeromonassens*, *Bacillus amyloliquefaciens*, *B. atrophaeus*, *B. licheniformis*	Acetic acid, itaconic acid, isobutyric acid, isovaleric acid, lactic acid	Mangrove	[177,183]
*Penicillium rugulosum*	Gluconic acid, citric acid	Venezuelan phosphate rocks	[184]
*Enterobacter intermedius*	2-ketogluconic acid	Grass rhizosphere	[185]
*Penicillium canescens*, *Aspergillus flavus*, *A. niger*	Citric acid, gluconic acid, oxalic acid, succinic acid	Wheat grains	[176,186]
*Pseudomonas fluorescens*	Tartaric acid, citric acid, malic acid, gluconic acid	Oil palm rhizosphere	[182,187]
*Aspergillus niger*	Gluconic acid, oxalic acid	Tropical and subtropical soils	[188]
*P. trivialis*	Lactic acid, formic acid	Rhizosphere *(Hippophae rhamnoides*) (Trans-Himalayas, cold Howl and Spiti deserts)	[177]
*Actinomadura oligospora; B. pumilus var.2*; *B. subtilis var.2*; *Citrobacter* sp.	Propionic acid, gluconic acid, isovaleric acid, caproic acid, heptonic acid, isocaproic acid, formic acid, valeric acid, succinic acid, oxalic acid, oxaloacetic acid, malonic acid	Giant cardon cactus (*P. pringlei*)	[189,190]
*B. pumilus CHOO8A; B. fusiformis*	Succinic acid, citric acid, gluconic acid, oxalic acid, 2-ketogluconic acid, lactic acid, malic acid, formic acid	*Opuntia cholla*	[177]
*Bacillus* sp. *SENDO 6*	Gluconic acid, isovaleric acid, propionic acid, lactic acid, formic acid, succinic acid	*P. pringlei*	[177]
*Bacillus megaterium M1PCa*, *Enterobacter sakazakii M2PFe*, *Pseudomonas putida M5TSA*	Acetic acid, formic acid, gluconic acid, lactic acid, oxalic acid, propionic acid, succinic acid	*Mammillaria fraileana* cactus	[173,191]
